# Prenatal healthcare after sentencing reform: heterogeneous effects for prenatal healthcare access and equity

**DOI:** 10.1186/s12889-022-13359-7

**Published:** 2022-05-12

**Authors:** Jaquelyn L. Jahn, Jessica T. Simes

**Affiliations:** 1 The Ubuntu Center On Racism, Global Movements and Population Health Equity, Drexel University Dornsife School of Public Health, 3600 Market St, Philadelphia, PA 19104 USA; 2 Department of Sociology, Boston University, 100 Cummington Mall, Boston, MA 02215 USA

**Keywords:** Incarceration, Prenatal care, Criminal justice reform, Health equity

## Abstract

**Background:**

High rates of imprisonment in the U.S. have significant health, social, and economic consequences, particularly for marginalized communities. This study examines imprisonment as a contextual driver of receiving prenatal care by evaluating whether early and adequate prenatal care improved after Pennsylvania’s criminal sentencing reform reduced prison admissions.

**Methods:**

We linked individual-level birth certificate microdata on births (*n* = 999,503) in Pennsylvania (2009–2015), to monthly county-level rates of prison admissions. We apply an interrupted time series approach that contrasts post-policy changes in early and adequate prenatal care across counties where prison admissions were effectively reduced or continued to rise. We then tested whether prenatal care improvements were stronger among Black birthing people and those with lower levels of educational attainment.

**Results:**

In counties where prison admissions declined the most after the policy, early prenatal care increased from 69.0% to 73.2%, and inadequate prenatal care decreased from 18.1% to 15.9%. By comparison, improvements in early prenatal care were smaller in counties where prison admissions increased the most post-policy (73.5 to 76.4%) and there was no change to prenatal care inadequacy (14.4% pre and post). We find this pattern of improvements to be particularly strong among Black birthing people and those with lower levels of educational attainment.

**Conclusions:**

Pennsylvania’s sentencing reforms were associated with small advancements in racial and socioeconomic equity in prenatal care.

**Supplementary Information:**

The online version contains supplementary material available at 10.1186/s12889-022-13359-7.

## Background

Approximately 1.2 million people enter or leave prisons in the United States each year, [1] representing a significant population-level dynamic in marginalized communities. Prison admissions are highly geographically concentrated within racially and economically segregated and communities across the urban–rural continuum [2–4]. Under these conditions of mass incarceration, whole communities have been harmed by the scale of the U.S. prison system, impacting not just those who have been policed and incarcerated, but also their families and broader social networks.

Incarceration has been widely examined as a structural determinant of racial and economic health inequities. Losing a partner or family member to imprisonment may cause significant psychological and financial burden for family members and loved ones, including shifting caretaking responsibilities and housing instability [5–7]. These hardships and interruptions can occur during sensitive developmental periods in the lives of those left behind, such as pregnancy and birth. Psychosocial and material stressors before and during pregnancy can adversely affect maternal and infant health and perinatal healthcare utilization [8–11]. Moreover, contact with the criminal legal system may deter individuals from engaging with other surveilling institutions including the healthcare system, [12] which presents another avenue through which incarceration could reduce prenatal care receipt.

Early and high-quality prenatal care have long been considered critical in promoting maternal health and preventing adverse birth outcomes [13, 14]. Recent studies have examined how individual barriers such as insurance status or unemployment, or structural barriers such as weak social supports, racism, and neighborhood inequality, influence preventative care utilization [15–18]. One study in an urban setting estimated 30–40% of the variance in women’s preventative care utilization is explained by neighborhood conditions [15]. In addition to direct experiences with family member incarceration, having more of one’s social network affected by incarceration or living in a community where many are economically strained by incarceration may also determine preventative care access and utilization by shaping, for example, one’s ability to secure reliable transportation or childcare [13, 19–21]. Area-level rates of imprisonment are also related to previously identified contextual predictors of insufficient prenatal care, including greater proportions of women-headed households, fewer married couples, and disrupted social support [15]. However, no prior literature has examined whether policies that have reduced rates of incarceration have spillover effects for prenatal care.

In the past decade, several states have started to reform harsh penalties that have contributed to mass incarceration [22]. We use Pennsylvania as a case for understanding the spillover effects of sentencing reforms for prenatal care receipt. In 2015, Pennsylvania had the seventh largest prison system in the country, with an incarceration rate of 394 per 100,000 residents, approximately the national average [23]. Pennsylvania sits right at the national average for prenatal care receipt, with 77 percent of pregnant people receiving prenatal care in the first trimester [24]. In 2012 Pennsylvania state lawmakers passed omnibus amendments to the crime, judicial, prison and parole code (Act 122 and Act 196), which sought to reduce prison admissions by limiting the number of people sent to prison for low-level violations and scaling back harsh mandatory minimum sentences [25, 26]. Reductions in prison populations and the resultant fiscal savings were partially “reinvested” in county-level reentry programs in an effort to reduce returns to prison [27]. In certain states these justice reinvestment initiative reforms led to a decline in prison admissions, [28] but how these changes may have affected communities and prenatal care is untested.

We designed a study using individual-level birth records from Pennsylvania (2009–2015) to test the following two hypotheses: First, we hypothesize that there will be gradual improvements in early and adequate prenatal care utilization after implementation of Pennsylvania’s criminal sentencing reform policy, but only in counties where prison admissions declined after the policy. Second, given that structural racism and socioeconomic marginalization make Black people and those with lower levels of education more exposed to the criminal legal system, we expect any effects of the policy to be stronger for these populations.

## Methods

### Study population

We use individual-level birth certificate microdata for all births in Pennsylvania (2009–2015) from the National Center for Health Statistics (NCHS). Our study examines two outcomes, early (first trimester) prenatal care and adequate prenatal care as measured using the Revised-Graduated Prenatal Care Utilization Index (R-GINDEX) [29]. Both outcome measures were constructed using data on month prenatal care began, gestational age, and number of prenatal visits from the birth certificate. We linked individual births with county-level attributes using birthing parent’s county of residence. County-level prison admissions data were provided by the Pennsylvania Department of Corrections. We also used data from the American Community Survey (7 years of ACS 5-year Estimates, 2009–2015) and the FBI Uniform Crime Reports (UCR). Note that throughout our manuscript we use gender inclusive language when discussing attributes of the birthing parent in our study population, given that birth certificate records do not include information on gender identity. The NCHS and Harvard Longwood Campus Institutional Review Board approved analysis of birth microdata. Our manuscript meets STROBE guidelines for reporting for observational studies.

### Measures

#### Individual-level

To address potential confounding, we obtained data on self-reported age (< 19, 20–29, 30–39, 40 + years), and marital status (married, unmarried) from NCHS (Table 1). Our analyses also examine effect heterogeneity across birthing person race/ethnicity and educational attainment. Birthing person race and ethnicity in the birth certificate data were self-reported and include the following categories: non-Hispanic Black, non-Hispanic White, non-Hispanic American Indian or Alaska Native, non-Hispanic Asian or Pacific Islander (hereafter referred to as “Black”, “White”, “American Indian & Alaska Native” and “Asian” for brevity) and Hispanic [30]. Educational attainment was self-reported and categorized as less than high school, high school or GED, and greater than high school.

**Table 1:** Characteristics of births in Pennsylvania, 2009–2015

	**PA** *N* = 999,503	**PA Pre-Policy** *N* = 642,713	**PA Post-Policy** *N* = 356,790
Prenatal care 1^st^ trimester Missing	711,431 (71.2%) 47,386 (4.7%)	449,983(70.0%) 31,783 (5.0%)	261,448(73.3%) 15,603 (4.4%)
Inadequate/no prenatal care Intermediate Adequate Intensive Missing	139,672 (14.0%) 395,932 (39.6%) 348,689 (34.9%) 42,358 (4.24%) 72,852 (7.3%)	92,755 (14.4%) 256,302 (39.9%) 216,933 (33.8%) 25,376 (4.0%) 51,347 (8.0%)	46,917 (13.1%) 139,630 (39.1%) 131,756 (36.9%) 16,982 (4.8%) 21,505 (6.0%)
Age			
< 19 20–29 30–39 40 + Missing	70,234 (7.0%) 504,862 (50.5%) 396,583 (39.7%) 27,824 (2.8%) 0	50,579 (7.9%) 327,159 (50.9%) 247,011 (38.4%) 17,964 (2.8%) 0	19,655 (5.5%) 177,703 (49.8%) 149,572 (41.9%) 9860 (2.8%) 0
Race/ethnicity			
Non-Hispanic White Non-Hispanic Black Hispanic American Indian & Alaska Native Asian & Pacific Islander Missing	700,537 (70.1%) 145,665 (14.6%) 99,651 (10.0%) 1483 (0.1%) 42,475 (4.3%) 9692 (1.0%)	452,299(70.4%) 94,440(14.7%) 62,699 (9.8%) 975 (0.2%) 26,578 (4.1%) 5722 (0.9%)	248,238 (69.6%) 51,225 (14.4%) 36,952 (10.3%) 508 (0.1%) 15,897 (4.4%) 3970 (1.1%)
Educational attainment			
< High school High school > High school Missing	139,940 (14.0%) 250,655 (25.1%) 599,468 (60.0%) 9440 (0.9%)	94,622 (14.7%) 161,667 (25.2%) 372,605 (59.0%) 379,645 (1.1%)	45,318 (12.7%) 88,988 (24.9%) 219,823 (61.6%) 2661 (0.8%)
Insurance status			
Medicaid Private Insurance Self-Pay Indian Health Service CHAMPUS/TRICARE Other Government Other Missing	317,573 (31.8%) 572,496 (57.3%) 49,711 (5.0%) suppressed (< .1%) 188 (< .1%) 138 (< .1%) 27,214 (2.7%) 32,181 (3.2%)	203,769 (31.7%) 366,343 (57.0%) 32,795 (5.1%) suppressed (< .1%) 102 (< .1%) 103 (< .1%) 18,614 (2.9%) 20,985 (3.3%)	113,804 (31.8%) 206,153 (57.8%) 16,916 (4.8%) suppressed (< .1%) 86 (< .1%) 35 (< .1%) 8600 (2.4%) 11,196 (3.1%)
Married	585,652 (58.6%)	376,125 (58.5%)	209,527 (58.7%)
Missing	0	0	0
County-level prison admissions per 1000 mean (SD)	0.12 (0.07)	0.13 (0.08)	0.12 (0.07)

#### County-level

We constructed monthly county-level rates of prison admissions per 100,000 population based on the county of commitment from court and denominator data from the American Community Survey. Rates were constructed excluding admissions with no associated geographic data (2% of the available data). We adjust for county-level annual crime rate given that crime could influence both the policy uptake and, independently, prenatal care utilization. We use crime data from the UCR, which includes Part-I crimes (i.e. criminal homicide, forcible rape, robbery, aggravated assault, burglary, larceny-theft, motor vehicle theft, and arson).

### Statistical analysis

We first examined bivariate and univariate distributions of our study’s exposures and outcomes before and after Pennsylvania’s criminal justice reform policy’s implementation (Table 1). We restrict our analysis to birth records without missing data (*n* = 901,838), given that missingness in our variables was relatively low (Table 1), the large sample size, and that our computational capacity precluded multiple imputation.

To evaluate possible changes in our study’s outcomes after Pennsylvania’s sentencing guideline reforms, we leverage geographic variation in the extent to which the policy was more effective in reducing prison admissions. We evaluate whether any post-policy improvements in prenatal care were larger in counties with the greatest declines versus continued increases in prison admission rates after the policy. Our interrupted time series models stratify across quartiles of county-level pre-post difference in mean monthly prison admission rates. We then examine effect heterogeneity across birthing person educational attainment and race/ethnicity, and hypothesize larger changes among groups likely to be more affected by incarceration in their social networks (i.e. those with < high school or high school degree vs. > high school degree; and non-Hispanic Black vs. White race/ethnicity). We estimate the change in average levels of prenatal care after the policy but emphasize the time trend slope across groups to: 1) assess whether improvements were distinct from rises that pre-dated the policy, and 2) address difficulties in determining the lag in policy implementation necessary to have a population-level effect on prenatal care. To do so we implemented Poisson regression models with robust error variance that interacted a post policy variable with a linear monthly time trend and, separately, our 3-category education variable and race/ethnicity variable. Models adjusted for individual-level race, age, marital status, insurance type, annual county-level crime rate, and included county-level fixed effects.

## Results

Around 70% of births in Pennsylvania received early (first trimester) prenatal care and about 14% received inadequate prenatal care between 2009–2015 (Table 1). However, there were notable disparities in prenatal care across levels of educational attainment and race/ethnicity. Just over half (51.9%) of those with less than high school education received prenatal care in the first trimester, whereas this number was 82.1% for those with greater than high school education, and similar gaps were observed for those who received inadequate prenatal care (30.5% vs. 10.7%). Black birthing people had lower levels of early (63.1%) and higher levels of inadequate (24.5%) prenatal care relative to White birthing people (early: 79.2%, inadequate: 12.1%). There were also differences in county-level prison admission rates across levels of education and race/ethnicity, with rates consistently highest among those with less than high school education and Black birthing people.

### Post-policy change in prenatal care across counties

After the sentencing reform policy was implemented, prison admissions did not decline uniformly across counties in Pennsylvania. Whereas admissions declined from 23.2 to 20.6 per 100,000 population in Philadelphia County, admissions increased in about half of the state’s counties. In counties where prison admissions declined the most after the policy (Q4), rates were reduced by 1.10–4.28 per 100,000. In these Q4 counties, early prenatal care increased from 69.0% to 73.2% of births, and inadequate prenatal care decreased from 18.1% to 15.9%. By comparison, in counties where prison admissions increased the most after the policy (Q1), rates increased by 1.34 to 10.6 per 100,000. In these Q1 counties, the increase in early prenatal care was smaller (73.5 to 76.4%) than in Q4 counties and there was no change to prenatal care inadequacy (14.4% pre and post).

### Effect heterogeneity across birthing person educational attainment

**Fig. 1:**
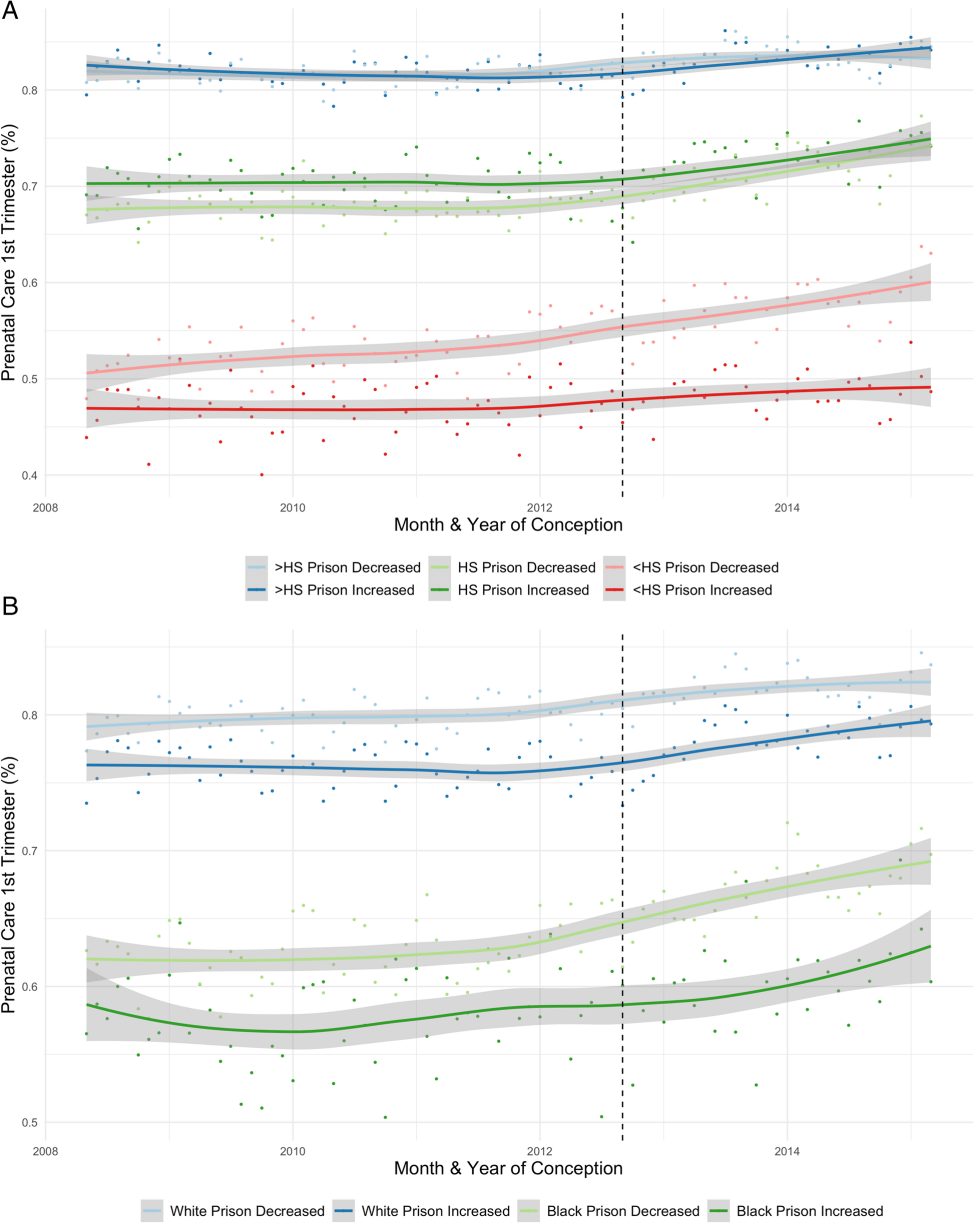
Early prenatal care in Pennsylvania counties where post-policy prison admissions increased and decreased across levels of education (**A**) and race/ethnicity (**B**) (2009–2015). Panel A: Average monthly rates of first trimester prenatal care in counties where prison admissions decreased and increased after the 2012 policy across birthing person levels of education, trend line estimated using a loess smoothing function (span = 0.75). Panel B: Average monthly rates of first trimester prenatal care in counties where prison admissions decreased and increased after the 2012 policy across birthing person race/ethnicity, trend line estimated using a loess smoothing function (span = 0.75)

Figure 1 Panel A describes the time trends in early prenatal care across birthing person educational attainment in counties where prison admissions increased and decreased. Our interrupted time series models suggest that within counties where prison admissions declined the most after the policy (Q4), early prenatal care improved most for those with a high school degree or less (Table 2: % increase < HS: 8.13% [95% CI: 6.35, 9.95%]; HS: 4.12% [95% CI: 3.02, 5.23%]; > HS: 0.80% [95% CI: 0.09, 1.52%]). These increases represented a significant rise in the time trend in early prenatal care after the policy reform for those with lower levels of educational attainment but not for those with greater than a high school degree (< HS slope increased by 0.22% [95% CI: 0.06, 0.37%]; HS: 0.23% [95% CI: 0.14, 0.32%]; > HS -0.03 [95% CI: -0.08, 0.02%]). These changes in the time trend for those with lower levels of education suggest improvements above and beyond steady increases in early prenatal care that began before the policy. By contrast, in counties where admissions increased the most (Q1), there were smaller average improvements across all levels of educational attainment, and the trend did not differ from pre-policy improvements (Table 2). Results in counties with moderate increases and decreases (Q2 and Q3) showed a similar pattern to findings from counties in Q1 and Q4 (Supplemental Tables 1 and 2).

**Table 2:** Estimated changes in early and inadequate prenatal care before and after the policy across birthing person education and race/ethnicity

Outcome	Effect heterogeneity	Average % change in pre vs. post policy (95% CIs)		% Change in pre-policy trend vs. post-policy trend (95% CIs)	
		Q1 County prison admissions increased the most	Q4 County Prison admissions decreased the most	Q1 County prison admissions increased the most	Q4 County Prison admissions decreased the most
First trimester prenatal care	Education				
	> High school High school < High school	**2.71 (1.62, 3.81)** **3.99 (2.29, 5.72)** **4.58 (0.73, 8.59)**	**0.80 (0.09, 1.52)** **4.12 (3.02, 5.23)** **8.13 (6.35, 9.95)**	0.00% (-0.10, 0.11) 0.10% (-0.06, 0.27) 0.06% (-0.31, 0.44)	-0.03% (-0.08, 0.02) **0.23% (0.14, 0.32)** **0.22% (0.06, 0.37)**
	Race/ethnicity				
	Non-Hispanic White Non-Hispanic Black	**2.97 (2.01, 3.93)** 6.14 (-1.97, 14.91)	**1.55 (0.83, 2.29)** **4.68 (3.22, 6.16)**	0.04% (-0.05, 0.13) 0.25% (-0.53, 1.03)	-0.01% (-0.06, 0.04) **0.23% (0.11, 0.35)**
Inadequate prenatal care	Education				
	> High school High school < High school	-6.64 (-13.04, 0.22) **9.20 (1.09, 17.96)** 5.25 (-0.02, 10.80)	**-17.92 (-20.56, -15.20)** **-7.06 (-10.11, -3.91)** **-3.74 (-6.89, -0.48)**	0.06% (-0.62, 0.76) 0.19% (-0.55, 0.95) -0.15% (-0.65, 0.35)	0.21% (-0.05, 0.48) **-0.42% (-0.68, -0.15)** **-0.28% (-0.56, -0.00)**
	Race/ethnicity				
	Non-Hispanic White Non-Hispanic Black	3.49 (-0.73, 7.88) -3.17 (-19.13, 15.93)	**-14.53 (-17.27, -11.71)** **-4.36 (-7.68, -0.93)**	-0.02% (-0.42, 0.38) 0.23% (-1.47, 1.95)	0.23% (-0.03, 0.51) **-0.37% (-0.62, -0.12)**

Average percent change in each outcome was estimated from Poisson models with robust error variance that interacted a post policy variable with birthing person education and, separately, race/ethnicity. The change in pre- versus post-policy trends in each outcome were estimated by interacting the post-policy variable with a linear monthly time trend and birthing person education and, separately, race/ethnicity. All models were stratified across quartiles of post-policy changes in county prison admissions, and adjust for age, marital status, insurance type, crime rate, and included county-level fixed effects. In Q1 counties, rates of prison admissions increased by 1.34–10.6 per 100,000, whereas in Q4 counties prison admissions decreased by 1.10 - 4.28 per 100,000

Moreover, in counties where admissions declined the most (Q4), inadequate prenatal care improved (decreased) across all levels of education, but the rate of decline was steeper after the policy only among those with high school education or less (< HS -0.28% decline in slope [95% CI: -0.56, -0.002]; HS -0.42 [95% CI:-0.68, -0.15%], > HS 0.21 [95% CI: -0.05, 0.48]). In counties where prison admissions increased after the policy (Q1), inadequate prenatal care worsened (increased) among those with high school education (Table 2). There were no strong changes in other prenatal care adequacy variables.

### Effect heterogeneity across birthing person race/ethnicity

We additionally examined changes in prenatal care for non-Hispanic Black and White birthing people across counties where the policy was and was not effective in reducing prison admissions. Figure 1 Panel B shows that early prenatal care improved after the policy for Black birthing people in counties where prison admissions declined after the policy. Our interrupted time series models (Table 2) estimated that on average, in counties where prison admissions decreased the most after the policy (Q4), early prenatal care improved by 4.68% (95% CI: 3.22, 6.16%) among Black birthing people, with smaller improvements among White birthing people (1.55%, 95% CI: 0.83, 2.29%). Indeed, when examining pre versus post policy changes in the trends in early prenatal care across these groups, the trend in early prenatal care for Black birthing people increased 0.23% (95% CI: 0.11, 0.35%) after the policy, but the change in the trend for White birthing people was not significant, suggesting that improvements among White birthing people were not different from steady increases that predated the policy. By contrast, in counties where prison admissions increased the most after the policy (Q1), there were no significant changes in prenatal care for Black birthing people. Although prenatal care improved for White birthing people (2.97%, 2.01, 3.93%), this improvement did not represent a departure from the pre-trend for this group (% change in trend: 0.04%, 95% CI: -0.05, 0.35%).

Similarly, when we modeled inadequate prenatal care in counties where prison admissions declined the most after the policy (Table 2), we observed improvements among both Black (-4.36, 95% CI: -7.68, -0.93%) and White birthing people (-14.53, 95% CI: -17.27, -11.71%). Importantly, although relative declines in inadequate care were larger among White people, there was no significant change in the trend for this group (0.23%, 95% CI: -0.03, 0.51%). Conversely, the improvements among Black birthing people represented a departure from the pre-policy trend in inadequate prenatal care (-0.37%, 95% CI: -0.62, -0.12%). In counties where prison admissions increased the most after the policy, there were no significant changes in inadequate prenatal care for either Black or White birthing people.

## Discussion

Our examination of the effects of Pennsylvania’s criminal sentencing reform showed that after the policy was implemented, early prenatal care increased on average and inadequate prenatal care declined. Our fixed effects interrupted time series design used multiple points of comparison to assess whether reductions in incarceration improved racial and socioeconomic health equity. First, we found the benefits for prenatal care were largely limited to counties where prison admission rates declined the most after the policy. Second, we found that improvements were primarily observed among groups that are more likely to be affected by prison admissions, Black birthing people and those with lower levels of education, thus decreasing prenatal care inequities across these dimensions. Both points of comparison bolster confidence in the conclusion that changes in prenatal care were due to the policy and not to secular trends that affected these groups equally.

These findings underscore the importance of contextual conditions of incarceration for preventative health care access and utilization. Prior research has largely examined individual or household-level effects of incarceration on prenatal care, [21] but prenatal care has not been examined in the prior epidemiologic literature on incarceration as a contextual effect across geographies. Moreover, previous research on incarceration as a contextual predictor of adverse birth outcomes [20, 31] has thus far not tested criminal justice reform policies as potential interventions to reduce exposure to high rates of incarceration in communities.

Our findings also shed light on how criminal justice reforms may have spillover effects for healthcare utilization and health equity. However, the uneven implementation of the policy across counties underscores that incremental changes to criminal justice policy are unlikely to have broad effects for health equity. Several factors likely contributed to the heterogeneous implementation of the Pennsylvania’s policy, including judicial discretion and adherence to the revised sentencing guidelines. Indeed, policies like the one in Pennsylvania have been critiqued for making a small or negligible reduction in incarceration rates, and for further investing in criminal justice institutions instead of community-based services [28]. Moreover, even in counties where prison admissions declined the most, the magnitude of many of these improvements was small.

### Limitations

Although we attempt to address other factors that could explain the trends in prenatal care after the policy using comparisons across race/ethnicity, educational attainment, and post-policy county-level changes in prison admissions, we were not able to compare these to a control state because 1) 34 other states undertook justice reinvestment initiatives around the same time but enacted different policy changes to address state-specific issues, [22] and 2) of the states that did not undertake justice reinvestment initiatives, many implemented other strategies to reduce incarceration rates during these years [32]. Our results therefore might not be generalizable outside of Pennsylvania. Additionally, unobserved changes to healthcare could also affect the causal interpretation of our interrupted time series results. However, our findings are unlikely to be biased by the Affordable Care Act Medicaid expansion provision, because we adjust for individual-level insurance status and Pennsylvania expanded Medicaid only in the last year of our study period, 2015. A final limitation is our measurement of home residence using the county of commitment—the only available geographic data in the Pennsylvania prison admissions data file. While we use county of court commitment to proxy home communities, one study found that approximately one-third of incarcerated people returned to a county that was different from their county of commitment [33]. The available data precluded analysis at lower geographic levels (e.g. census tract) for which stronger effects of the policy might be observed.

## Conclusions

Our findings demonstrate the importance of analyzing incarceration as a contextual-level determinant of preventative healthcare, specifically prenatal care for racially and socioeconomically marginalized groups. In a period of significant criminal justice policy reform across the U.S., our findings suggest that incremental reductions in prison admissions will likely only have small impacts for prenatal care equity. We believe widely-implemented, transformative policy changes in the areas of healthcare, social welfare, and criminal justice together will be necessary to see dramatic shifts in preventative healthcare inequities.

## Supplementary Information

**Additional file 1. Supplemental Figure 1: **Inadequate prenatal care in Pennsylvania counties where post-policy prison admissions increased and decreased across levels of education (A) and race/ethnicity (B) (2009-2015). **Supplemental Table 1: **Changes in early and inadequate prenatal care before and after the policy across birthing person education and race/ethnicity. **Supplemental Table 2: **Changes in early and inadequate prenatal care before and after the policy across birthing person education and race/ethnicity.

## Data Availability

This study used restricted access birth certificate microdata from the U.S. National Center for Health Statistics. Researchers can apply for access to these data for research purposes at https://www.cdc.gov/nchs/nvss/nvss-restricted-data.htm. Prison admissions data were acquired through Data Use Agreement from the Pennsylvania Department of Corrections and are not publicly available. Researchers can inquire about accessing the data with the Pennsylvania Department of Corrections.
