# Supplementary material for: Prenatal healthcare after sentencing reform: heterogeneous effects for prenatal healthcare access and equity
Source: BMC Public Health. 2022 May 12;22:954. doi: 10.1186/s12889-022-13359-7 (PMC9102339; doi:10.1186/s12889-022-13359-7)
Supplement: Supplementary file 1 — Additional file 1. Supplemental Figure 1: Inadequate prenatal care in Pennsylvania counties where post-policy prison admissions increased and decreased across levels of education (A) and race/ethnicity (B) (2009-2015). Supplemental Table 1: Changes in early and inadequate prenatal care before and after the policy across birthing person education and race/ethnicity. Supplemental Table 2: Changes in early and inadequate prenatal care before and after the policy across birthing person education and race/ethnicity. [file 12889_2022_13359_MOESM1_ESM.docx]

**Supplemental Figure 1: Inadequate prenatal care in Pennsylvania counties where post-policy prison admissions increased and decreased across levels of education (A) and race/ethnicity (B) (2008-2015)**

**A B**


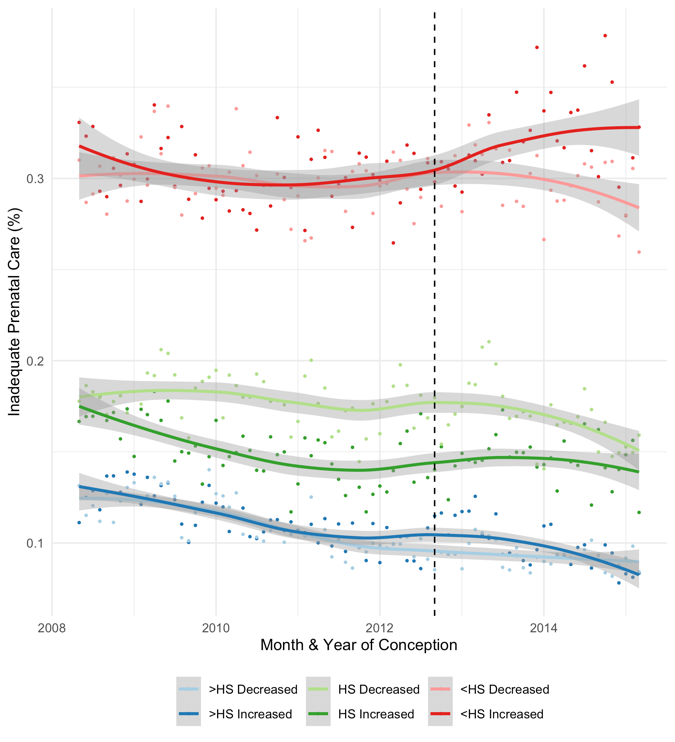

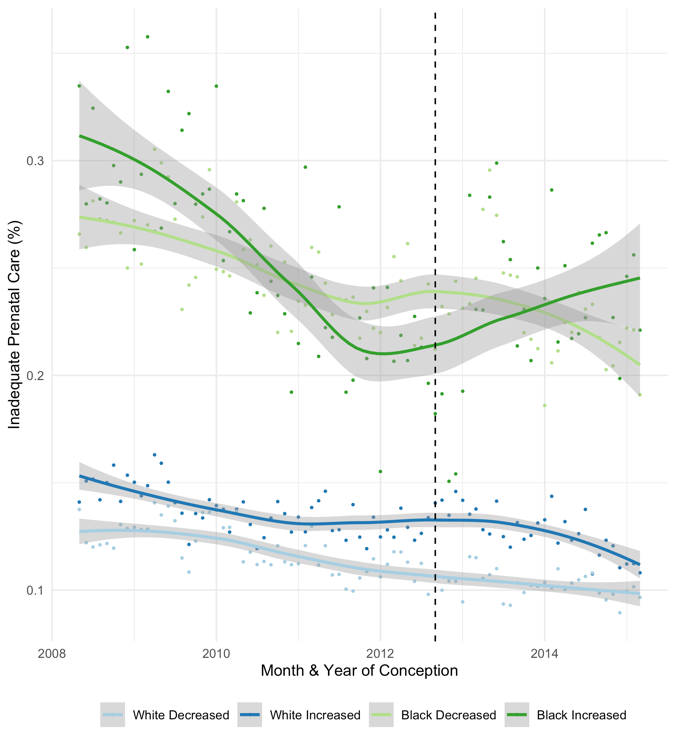


Panel A: Average monthly rates of inadequate prenatal care in counties where prison admissions decreased and increased after the 2012 policy across levels of education, trend line estimated using a loess smoothing function (span=0.75). Panel B: Average monthly rates of inadequate prenatal care in counties where prison admissions decreased and increased after the 2012 policy across birthing person race/ethnicity, trend line estimated using a loess smoothing function (span=0.75).

**Supplemental Table 1:** Changes in early and inadequate prenatal care before and after the policy across birthing person education and race/ethnicity

| Outcome | Effect heterogeneity | Average % change in pre vs. post policy (95% CIs) | | | |
| --- | --- | --- | --- | --- | --- |
|  |  | Q1  County prison admissions increased by 1.34 to 10.60 per 100,000 | Q2  County prison admissions increased by 0.02 to 1.13 per 100,000 | Q3  County Prison admissions decreased by 0.01 to 0.86 per 100,000 | Q4  County Prison admissions decreased by 1.10 to 4.28 per 100,000 |
| First trimester prenatal care | Education  >High school  High school  <High school | 2.71 (1.62, 3.81)  3.99 (2.29, 5.72)  4.58 (0.73, 8.59) | 0.75 (0.16, 1.34)  2.84 (1.70, 4.00)  5.71 (3.32, 8.16) | 1.28 (0.79, 1.78)  3.42 (2.38, 4.48)  4.24 (2.05, 6.48) | 0.80 (0.09, 1.52)  4.12 (3.02, 5.23)  8.13 (6.35, 9.95) |
|  | Race/ethnicity  Non-Hispanic White  Non-Hispanic Black | 2.97 (2.01, 3.93)  6.14 (-1.97, 14.91) | 1.44 (0.86, 2.02)  1.81 (-0.59, 4.26) | 1.37 (0.85, 1.88)  3.00 (1.70, 4.32) | 1.55 (0.83, 2.29)  4.68 (3.22, 6.16) |
| Inadequate prenatal care | Education  >High school  High school  <High school | -6.64 (-13.04, 0.22) 9.20 (1.09, 17.96)  5.25 (-0.02, 10.80) | -10.85 (-13.89, -7.70)  -3.08 (-7.08, 1.09) 8.15 (4.09, 12.37) | -8.83 (-11.93, -5.64)  1.78 (-2.92, 6.70)  12.86 (8.40, 17.51) | -17.92 (-20.56, -15.20)  -7.06 (-10.11, -3.91)  -3.74 (-6.89, -0.48) |
|  | Race/ethnicity  Non-Hispanic White  Non-Hispanic Black | 3.49 (-0.73, 7.88)  -3.17 (-19.13, 15.93) | -5.60 (-8.35, -2.78)  -1.27 (-6.36, 4.10) | -3.61 (-6.50, -0.62)  1.09 (-5.09, 7.67) | -14.53 (-17.27, -11.71)  -4.36 (-7.68, -0.93) |

Average percent change in each outcome was estimated from Poisson models with robust error variance that interacted a post policy variable with birthing person education and, separately, race/ethnicity. All models were stratified across quartiles of post-policy changes in county prison admissions, and adjust for age, marital status, insurance type, crime rate, and included county-level fixed effects.

**Supplemental Table 2:** Changes in early and inadequate prenatal care before and after the policy across birthing person education and race/ethnicity

| Outcome | Effect heterogeneity | % Change in pre-policy trend vs. post-policy trend (95% CIs) | | | |
| --- | --- | --- | --- | --- | --- |
|  |  | Q1  County prison admissions increased by 1.34 to 10.60 per 100,000 | Q2  County prison admissions increased by 0.02 to 1.13 per 100,000 | Q3  County Prison admissions decreased by 0.01 to 0.86 per 100,000 | Q4  County Prison admissions decreased by 1.10 to 4.28 per 100,000 |
| First trimester prenatal care | Education  >High school  High school  <High school | 0.00% (-0.10, 0.11)  0.10% (-0.06, 0.27)  0.06% (-0.31, 0.44) | 0.06% (0. 01, 0.11)  0.15% (0.04, 0.26)  -0.00% (-0.23, 0.23) | -0.06% (-0.10, -0.03)  0.04% (-0.06, 0.14)  -0.06% (-0.27, 0.15) | -0.03% (-0.08, 0.02)  0.23% (0.14, 0.32)  0.22% (0.06, 0.37) |
|  | Race/ethnicity  Non-Hispanic White  Non-Hispanic Black | 0.04% (-0.05, 0.13)  0.25% (-0.53, 1.03) | 0.07% (0.01, 0.12)  0.16% (-0.07, 0.39) | -0.02% (-0.06, 0.02)  -0.18% (-0.30, -0.06) | -0.01% (-0.06, 0.04)  0.23% (0.11, 0.35) |
| Inadequate prenatal care | Education  >High school  High school  <High school | 0.06% (-0.62, 0.76)  0.19% (-0.55, 0.95)  -0.15% (-0.65, 0.35) | -0.46% (-0.78, -0.13)  0.29% (-0.12, 0.70)  0.49% (0.13, 0.86) | 0.43% (0.12, 0.74)  -0.73% (-1.18, -0.29)  -0.32% (-0.68, 0.04) | 0.21% (-0.05, 0.48)  -0.42% (-0.68, -0.15)  -0.28% (-0.56, -0.00) |
|  | Race/ethnicity  Non-Hispanic White  Non-Hispanic Black | -0.02% (-0.42, 0.38)  0.23% (-1.47, 1.95) | -0.44% (-0.71, -0.17)  1.51% (1.00, 2.03) | 0.14% (-0.11, 0.39)  -0.33% (-0.93, 0.27) | 0.23% (-0.03, 0.51)  -0.37% (-0.62, -0.12) |

The change in pre- versus post-policy trends in each outcome were estimated by interacting the post-policy variable with a linear monthly time trend and birthing person education and, separately, race/ethnicity. All models were stratified across quartiles of post-policy changes in county prison admissions, and adjust for age, marital status, insurance type, crime rate, and included county-level fixed effects.
